# Parasitological, Molecular, and Histopathological Investigation of the Potential Activity of Propolis and Wheat Germ Oil against Acute Toxoplasmosis in Mice

**DOI:** 10.3390/pharmaceutics15020478

**Published:** 2023-02-01

**Authors:** Ashraf Mohamed Barakat, Khaled A. Abd El-Razik, Hassan Ali Mohamed El Fadaly, Walaa M. Saleh, Fatma Abo Zakaib Ali, Asmaa Aboelabbas Gouda, Sabry A. S. Sadek, Naief Dahran, Manal F. El-khadragy, Ehab Kotb Elmahallawy

**Affiliations:** 1 Department of Zoonotic Diseases, National Research Centre, El Buhouth St., Dokki, Giza 12622, Egypt; 2 Department of Animal Reproduction, Veterinary Research Institute, National Research Centre, Giza 12556, Egypt; 3 Department of Parasitology, Faculty of Veterinary Medicine, Sohag University, Sohag 82524, Egypt; 4 Department of Pathology and Clinical Pathology, Faculty of Veterinary Medicine, Sohag University, Sohag 82524, Egypt; 5 Department of Parasitology, Faculty of Veterinary Medicine, Zagazig University, Zagazig 44511, Egypt; 6 Department of Anatomy, Faculty of Medicine, University of Jeddah, Jeddah 21959, Saudi Arabia; 7 Department of Biology, College of Science, Princess Nourah bint Abdulrahman University, P.O. Box 84428, Riyadh 11671, Saudi Arabia; 8 Department of Zoonoses, Faculty of Veterinary Medicine, Sohag University, Sohag 82524, Egypt

**Keywords:** wheat germ, propolis, toxoplasmosis, liver, lungs, parasite load, tissue cyst, parasite burden

## Abstract

Toxoplasmosis is one of the most common parasitic zoonoses that affects all vertebrates. The drugs most commonly used against toxoplasmosis have many side effects, making the development of new antiparasitic drugs a big challenge. The present study evaluated the therapeutic effectiveness of novel herbal treatments, including propolis and wheat germ oil (WGO), against acute toxoplasmosis. A total of 50 albino mice were divided into five groups: group 1 (G1) (non-infected and non-treated); group 2 (G2) (infected without treatment); group 3 (G3) (treated with propolis); group 4 (G4) (treated with WGO); group 5 (G5) (treated with a combination of propolis and WGO). The effects of the herbal substances on different organs, mainly liver, spleen, and lungs, were investigated using parasitological, molecular, and histopathological examinations. The results of parasitological examination demonstrated statistically significant (*p* < 0.05) differences in the parasitic load between treated groups (G3, G4, and G5) compared to the control positive group (G2). These differences were represented by a significant reduction in the parasite load in stained tissue smears from the liver obtained from the animals treated with propolis (G3) compared to the parasite load in the positive control group. Similarly, animals (G4) treated with WGO exhibited a significant reduction in the parasite load versus the positive control group, while the lowest parasite load was found in G5, treated with propolis and WGO. Quantification of the parasite burden through molecular methods (PCR) revealed similar findings represented by reduction in the parasite burden in all treated groups with WGO and propolis as compared to the control group. Importantly, these previous parasitological and molecular findings were accompanied by a marked improvement in the histopathological picture of the liver, spleen, and lungs. In conclusion, propolis and WGO showed a good combination of therapeutic efficacy against acute toxoplasmosis.

## 1. Introduction

*Toxoplasma gondii* (*T. gondii*) is an intracellular zoonotic protozoan parasite that can affect all vertebrates, including humans and mammals [1]. The parasite has a complex life cycle consisting of a sexual cycle that occurs in felines as a definitive host and another asexual cycle in other mammals as an intermediate host [2]. Two forms of the parasites could be recognized in tissue: rapidly proliferated tachyzoite in the acute stage and dormant bradyzoite in the chronic stage [3]. The bradyzoites are able to encyst mostly in brain and muscle tissue [4,5]. The oocysts are developed within approximately one week inside the body; then, their shedding lasts 1–2 weeks after infection [6]. Humans can be infected in many ways, such as eating of undercooked meat containing bradyzoites [7], ingestion of contaminated water or vegetables infected with cat feces (sporozoite), or transmission of tachyzoites through the placenta from infected mother to fetus during pregnancy [8,9]. Following the cysts, containing bradyzoites, or oocysts, containing spores, are ingested, they reach the stomach and pass into the intestine, which stimulates innate immunity [10]. They cross the intestinal barrier, reaching the lymph nodes then the spleen, and are then distributed by the blood to other organs, such as the brain, liver, lungs, and heart. Although most toxoplasmosis may be asymptomatic, the immunosuppressed individuals experience a marked clinical picture, such as focal lesions in the brain causing encephalitis and neurological manifestations, followed by cyst formation in the tissue, blindness (ocular toxoplasmosis), abortion and neonatal death [11]. In livestock, mainly sheep, toxoplasmosis might result in a series of manifestations, such as abortion, stillbirth, and neonatal deaths, among others [12].

Diagnosis of toxoplasmosis mainly relies on the serological detection of tachyzoite antigens [13] or the detection of *T. gondii* DNA by PCR or examination of tachyzoites in stained tissue sections or body fluid smears, histologically, particularly during acute infection [14]. Real-time PCR (RT-PCR) is considered one of the most sensitive and specific molecular methods for the diagnosis of toxoplasmosis and the quantification of the parasite load. Several previous studies used RT-PCR to evaluate the herbal extract efficacy through quantification of parasite loads by targeting the p29 (GRA7) gene [15] in different treated groups [16,17,18]. Meanwhile, treatment of toxoplasmosis usually depends on using some chemical drugs, such as the pyrimethamine–sulfadiazine combination [19]. However, this combination is toxic and negatively affects the bone marrow, resulting in immunosuppression [20]. Numerous studies have revealed that spiramycin is a good alternative treatment with lower toxicity, but it poorly crosses the blood–brain barrier. The use of spiramycin is a promising alternative treatment method as it can reach at a high concentration in the placenta, giving it a high potential effect, especially against acute toxoplasmosis. Moreover, the existence of antiparasitic resistance makes developing new antiparasitic drugs challenging. Furthermore, due to the long-term use of these drugs, parasites became resistant [21]. It is, therefore, not surprising to state that these drugs are of limited use, especially during pregnancy, due to their side effects [22]. Therefore, there is an increasing demand for local medicinal herbs. In this respect, during the last decades, numerous in vitro and in vivo studies have demonstrated that plant extracts are a good alternative treatment against some protozoans, such as *Trypanosoma* spp. and *Leishmania* spp., with less side effects [23,24,25]. 

Regarding toxoplasmosis, various plant extracts, such as *Zingiber ofcinale* [26], *Eurycoma longifolia* [27], and propolis [28], have been proven to be effective against *T. gondii.* propolis is a natural bee product composed of approximately 300 compounds, including wax, essential oils, amino acids, and major phenolic compounds, such as flavonoids, in addition to some acids, such as caffeic acid [29]. Propolis showed broad-spectrum biological, anti-inflammatory, and antioxidant activities [30]. Furthermore, the hydroalcoholic extract of different types of propolis has antiparasitic effects against *Giardia lamblia* [31], *Toxoplasma gondii*, and *Trichomonas vaginalis* [32], where these extracts form a physical barrier against the parasite and inhibit the enzymes needed for host cell invasion. A previous study [33] reported that propolis inhibits *Toxoplasma* infection by suppressing tachyzoite multiplication. Additionally, using propolis at a high concentration leads to significant improvement in the histological shape of the infected mouse spleen [34]. Wheat germ oil (WGO) is considered a natural phytogenic feed additive commonly used as a food supplement for humans [35] and has a positive effect on general health, as it regulates serum lipid concentrations, decreases cholesterol absorption, enhances fertility, and delays aging [36].

Furthermore, WGO has anti-inflammatory, antioxidant [37,38], and antiparasitic properties [39,40]. A previous study reported that WGO has a significant therapeutic effect against *Cryptosporidium* [41], and another study recorded that it negatively affects the motility and growth of *Trichomonas vaginalis* trophozoites [42]. Our group revealed the potential activity of WGO and propolis against chronic toxoplasmosis in mice by reducing the parasite burden and restoring the observed histopathological changes in various organs [17,18]. However, no reliable information is available about the potential use of WGO and propolis and their combination against the acute form of the disease. The present study investigated the therapeutic effectiveness of herbal treatments, mainly propolis and WGO, in treating acute toxoplasmosis in mice through quantification of the parasite load by parasitological and molecular methods (PCR) followed by reporting the histological changes in liver, spleen and lungs. 

## 2. Materials and Methods

### 2.1. Materials Preparation and Chemical Characterization

Propolis extract (0.1 mL/day) and WGO (0.2 mg/1.5 mL/kg bw/day) were purchased from a local company for medicinal plants and herbs in Cairo Governorate, Egypt. Both substances were characterized using GC-MS analysis of silylated metabolites and metabolite identification as described elsewhere [18,43,44,45].

### 2.2. Animals

A total of 50 laboratory-bred female Swiss albino mice (6 weeks old, weighing 25–30 g) were used in the present study. Animals belonging to each group were divided and placed in a clean, well-ventilated separate cage with optimum climatic conditions and supplied with food and water ad libitum.

### 2.3. Treatment Protocol

Animals were acclimatized for one week before the experiment. At the age of 7 weeks, all animals were experimentally infected by *Toxoplasma* M49 strain (Avirulent strain) using 10^3^ cysts of previously infected mice, as described elsewhere [46,47]. Herbal treatment started on the second day after the infection for 10 days orally by a gastric tube, as mentioned in several previous studies [47,48]. Animals were regularly examined for any clinical signs, and their feces were also examined to exclude the presence of any other parasitic infections or bacteria [49,50]. Animals were grouped into five groups (10 mice per each), which included 40 infected animals, and 10 remained uninfected. The experimental protocol and groups are shown in Table 1 as follows: 

### 2.4. Parasitological Examination

One day after the end of the treatment, mice were sacrificed, and major parts of their livers were prepared for parasitological examination [53,54,55]. Tissues were dissected and homogenized with 1 mL saline in a tissue homogenizer (Wheaton, IL, USA). A total of 0.1 mL of each tissue suspension was placed on a slide to count the number of tissue cysts in 10 high-power fields (HPF). The mean number of tissue cysts, expressed as average parasite load in mL/liver homogenate, was measured for each animal, followed by calculating the mean number of tissue cysts for each infected group [56].

### 2.5. Histopathological Examination

At the end of the experimental period, animals were sacrificed, and tissue specimens from the liver (1–2 mg), spleen, and lungs were collected and fixed in 10% neutral-buffered formalin (NBF). Tissue specimens were prepared for staining by embedding them in paraffin wax blocks, sectioned to a thickness of 3–5 μm, and then stained with hematoxylin and eosin (H&E) [57], followed by performing a histopathological examination and cyst detection.

### 2.6. Histopathological Scoring

Each animal was scored according to the recorded histopathological examination [58]. A visual field inspection of at least 10 tissue specimen sections from each experimental group was performed to record the quantitative, semiquantitative, and histopathological changes. The number of parasitic cysts in each examined tissue was also counted in 10 randomized areas (approximately each 1 mm^2^), as described in [59,60]. Photographs were obtained with a 40× magnification lens. Tissue alterations were scored according to the following criteria: 1, 2, 3, and 4 (absent, mild, moderate, and severe, respectively) [61]. Liver sections were analyzed to detect the number of parasites present and the number of inflammatory foci [62]. Spleen tissue sections were scored according to the presence of the number of parasites, number of megakaryocytes, depletion of lymphocytes in the white and red pulp, and hemorrhage [63]. Lung tissue sections were scored according to alterations in bronchioles, alveolar structure, and hemorrhage. All analyses were performed by two researchers. The nature and extent of the lesion and its frequency of occurrence in randomly selected sites in the tissue [16,64].

### 2.7. Molecular Identification

The liver tissue (20–25 mg) of the experimental animals was prepared for DNA extraction using a GF-1 Tissue DNA extraction kit (Cat.-No. GF-TD-050, Vivantis Co., Shah Alam, Malaysia) following the manufacturer’s instructions. Real-time polymerase chain reaction (RT-PCR) was conducted using ViPrime PLUS Taq qPCR Green Master Mix I (SYBR^®^ Green Dye, Cat QLMM12 Vivantis Co., Malaysia) according to the manufacturer’s instructions. The primer sequences were designed using Laser gene DNA star software V15 (Table 2). Then, 2 μL of the extracted DNA was mixed with 10 μL (2X) PCR master mix and 0.1 μL (50 nmol) of each designed forward and reverse primer targeting the P29 gene. The amplification of the targeted gene (P29) was performed for 2 min at 95 °C, followed by 40 cycles of 95 °C/30 s and 60 °C/1 min using an MX30005P Agilent Real-Time PCR System (Agilent Technologies Deutschland GmbH, Waldbronn, Germany). The cycle threshold (CT) was determined when the fluorescence of a given sample significantly exceeded the baseline signal. A lower CT value represents a higher parasite load (DNA) and vice versa, while the negative CT represents the complete absence of the parasite.

### 2.8. Statistical Analysis

All data were entered into an Excel spreadsheet (Microsoft^®^ office 2013) and then exported to SPSS software (version 15.0) for statistical analysis. The mean and ± standard deviation (SD) were calculated, and the analysis of variance (ANOVA) between groups was calculated using a t-test. Histopathological scoring results were analyzed through GraphPad Prism, version 5 (San Diego, CA, USA). This software was used to compare the statistical significance between treatment groups through a one-way ANOVA with Tukey’s post-hoc multiple comparison tests. The statistical significance was considered to be *p* < 0.05.

## 3. Results

### 3.1. Parasitological Estimation of Parasite Load

**Figure 1:**
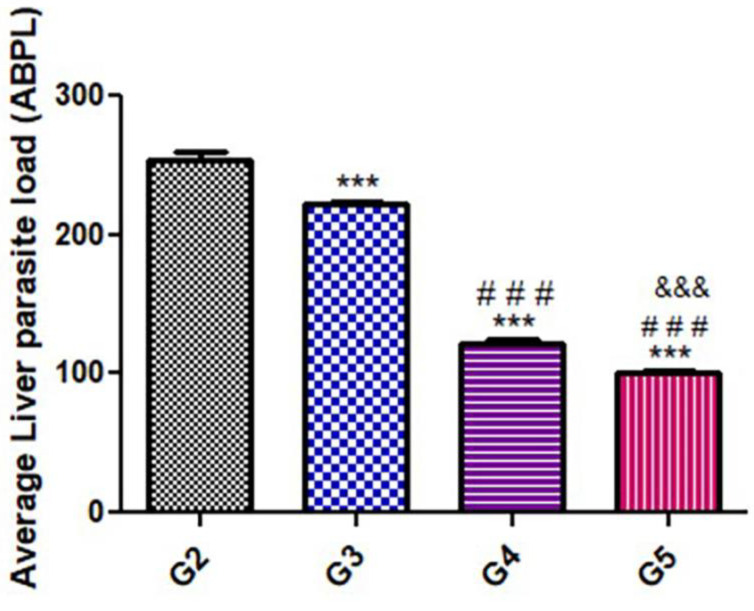
Average Liver parasite load (ABPL) in 1 mL/liver homogenate of treated mice as compared with untreated mice during the acute phase. Significant differences (G2) vs. other groups are marked by asterisks), (G3 vs. G4 and G5 are marked by #), ((G4 vs. G5 are marked by &) were measured using a one-way ANOVA with Tukey’s post hoc test: ***, ###, &&& *p* ≤ 0.001).

As shown in Figure 1, there were significant (*p* < 0.05) statistical differences in the parasitic load and the number of the counted cysts among the treated groups. There was a significant decrease in parasite load in the groups treated with the herbal substances (G3, G4, and G5) compared to the positive control group (G2).

### 3.2. Molecular Results

The DNA concentration of the *Toxoplasma P29* gene was quantified after treatment. As depicted in Table 3 and Table 4, all tested samples gave positive results with clear variations in the product quantities. Following statistical analysis, there was only a significant difference between the control untreated group (G2) and the group that was treated with a combination of WGO and propolis (G5).

### 3.3. Histopathological Evaluation

**Figure 2:**
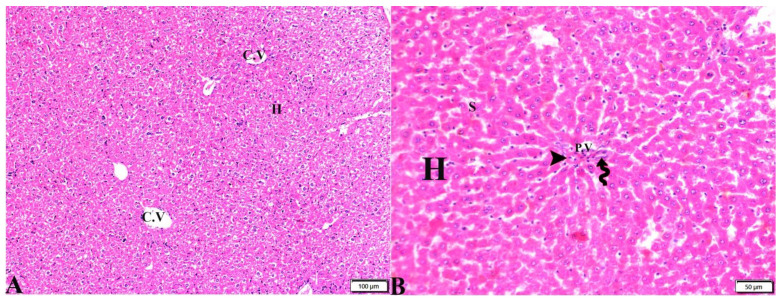
Photomicrograph of mice liver sections from control negative group (G1) demonstrating: (**A**) Normal hepatic architecture compromising in a normal central vein (C.V), sinusoids (S), and hepatocytes (H). (**B**) Normal portal triad structures: hepatic artery (arrowhead), portal vein (P.V), and bile duct (zigzag arrow). H&E stain, the bar size is indicated under pictures.

**Figure 3:**
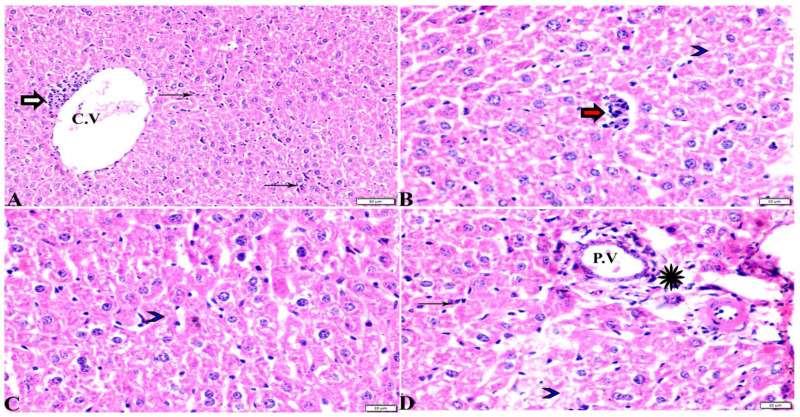
Photomicrograph of liver tissue sections from mice of *Toxoplasma gondii*-infected group (G2) showing (**A**–**D**): (**A**) Sever dilatation in the central vein (C.V), paracentral focal mononuclear cellular infiltration (white arrows), and interstitial inflammatory cellular infiltration (thin arrows). (**B**) Tissue cyst and bradyzoites (red arrows) in between hepatic tissues, necrotic hepatocytes (arrowhead). (**C**) Focal areas of hepatocellular necrosis (arrowhead). (**D**) Proliferative reaction of Kupffer cells (thin arrows), periportal edema (Star), markedly around portal vein (P.V), necrotic hepatocytes (arrowhead). H&E stain, the bar size is indicated under pictures.

**Figure 4:**
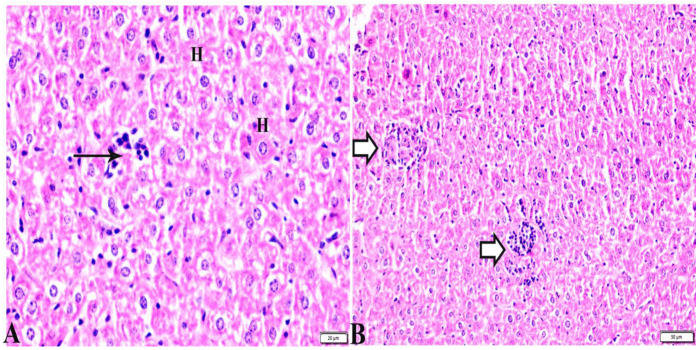
Photomicrograph of liver tissue sections from *Toxoplasma gondii*-infected group (G3) treated with propolis showing: (**A**) improvement in hepatocellular histologic structure (H), tachyzoites (arrows) in between hepatic tissues. (**B**) Multiple tissue cysts and their bradyzoites (white arrows). H&E stain, the bar size is indicated under pictures.

**Figure 5:**
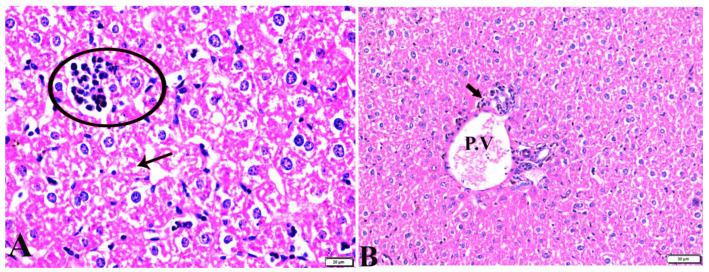
Photomicrograph of liver tissue sections from mice of *Toxoplasma gondii*-infected group (G4) treated with wheat germ oil showing: (**A**) Collection of bradyzoites in hepatocytes (circle), and focal areas of coagulative hepatocellular necrosis (arrow). (**B**) Dilated portal vein (P.V), mild periportal inflammatory cellular reaction (arrow). H&E stain, the bar size is indicated under pictures.

**Figure 6:**
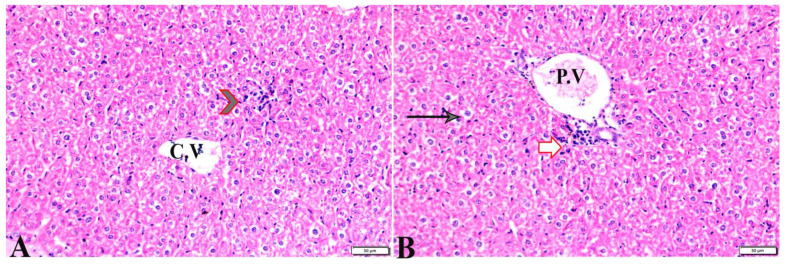
Photomicrograph of liver tissue sections from mice in the combination (propolis and Wheat germ)-treated group (G5) showing: (**A**) normal central vein (C.V), focal mononuclear cellular aggregation ((**B**), arrowheads) in between hepatic tissues. (**B**) mild vacuolar degeneration in hepatocytes structure (arrows), normal portal vein (P.V), periportal mononuclear cellular infiltration (white arrow). H&E stain, the bar size is indicated under pictures.

**Figure 7:**
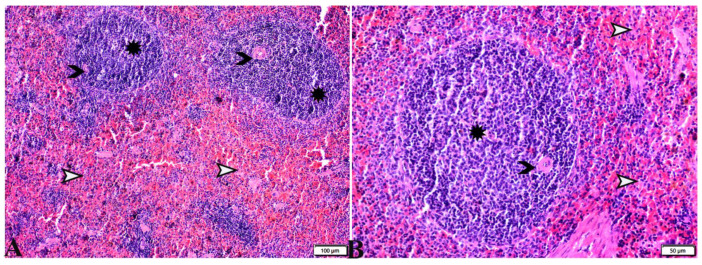
Photomicrograph of mice spleen sections from control negative group (G1) (**A**,**B**), demonstrating: Normal histological architecture of spleen; normal splenic artery (black arrowhead); normal sized white pulp with distinct cellular density in the germinal center (star); normal red pub (white arrowhead). H&E stain, the bar size is indicated under pictures.

**Figure 8:**
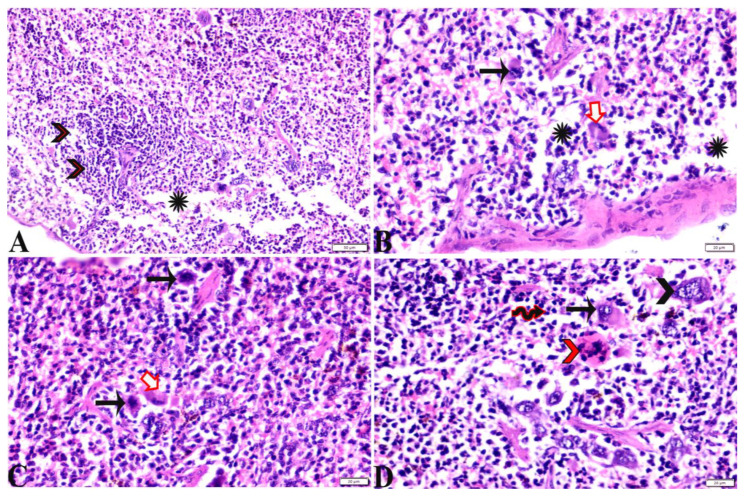
Photomicrograph of spleen tissue sections from *Toxoplasma gondii*-infected group (G2) showing: Severe depletion in lymphocyte population in splenic white pulp ((**A**), arrowheads) and the red pulp ((**A**,**B**), stars); the presence of marked numbers of megakaryocytes ((**B**–**D**), black arrows) between multiple bradyzoites. A single *Toxoplasma gondii* tachyzoite is also present ((**B**,**C**), white arrows). Multiple *Toxoplasma gondii* bradyzoites ((**D**), red arrowheads). Diffuse mononuclear leucocytic cellular infiltration ((**D**), zigzag arrows). H&E stain, the bar size is indicated under pictures.

**Figure 9:**
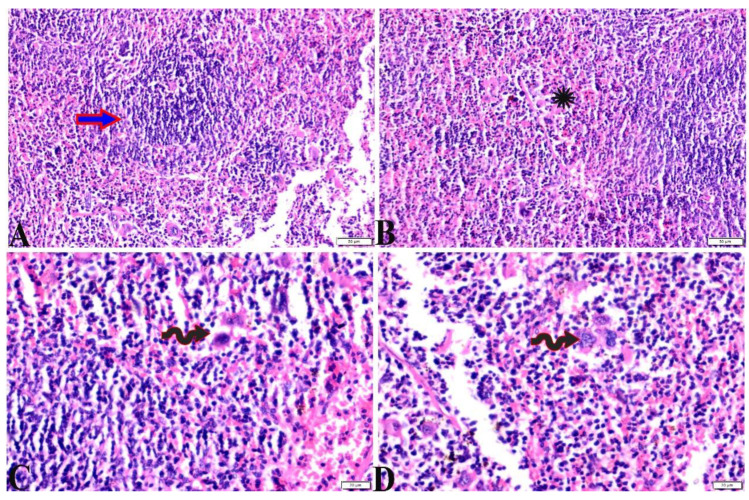
Photomicrograph of spleen tissue sections from *Toxoplasma gondii*-infected group (G3) treated with propolis showing: near normal sized splenic white pulp ((**A**), blue arrow), hemorrhage in the red pulp ((**B**), star), presence of some megakaryocytes ((**C**,**D**), zigzag arrows) surrounded by multiple bradyzoites. H&E stain, the bar size is indicated under the pictures ((**A**,**B**) = 50 µm, (**C**,**D**) = 20 µm).

**Figure 10:**
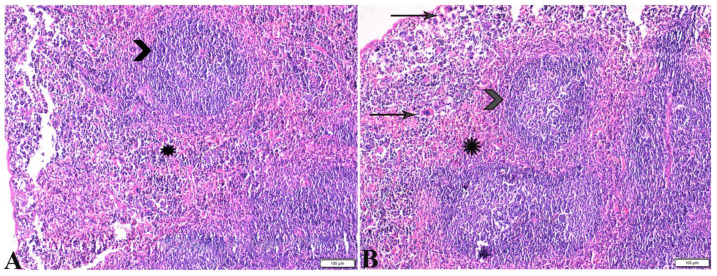
Photomicrograph of spleen tissue sections (**A**) from *Toxoplasma gondii* infected group treated with wheat germ oil (G4) showing: normal sized splenic white pulp (arrowheads), normal red pulp (star). (**B**) spleen tissue sections from combination (Propolis and Wheat germ) treated group (G5) showing: normal sized splenic white pulp (arrow heads), normal red pulp (stars). Presence of some megakaryocytes (thin arrows). H&E stain, the bar size was indicated under pictures.

**Figure 11:**
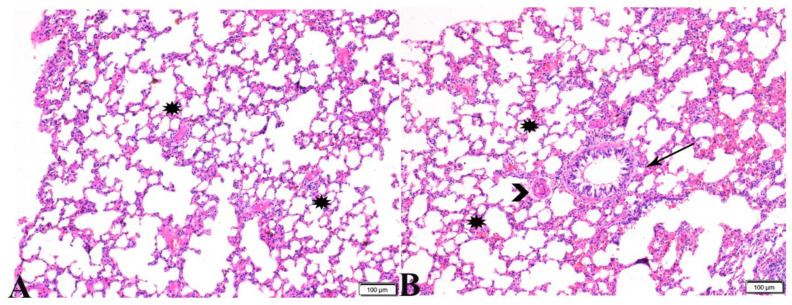
Photomicrograph of lung tissue sections from negative control group (G1) showing: (**A**,**B**) Normal alveolar tissue (stars). (**B**) Normal bronchiolar structure (arrows), and normal vasculature (arrowhead). H&E stain, the bar size is indicated under pictures.

**Figure 12:**
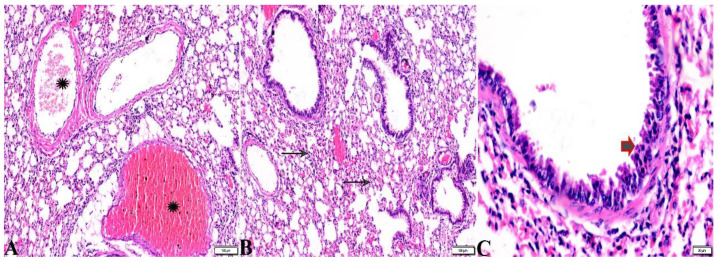
Photomicrograph of lung tissue sections from *Toxoplasma gondii*-infected group (G2) showing: ((**A**), magnified in (**B**,**C**)) congested blood vessels (stars), atelectasis in alveoli with mild interstitial hemorrhage ((**B**), arrows), hyperplasia in the bronchial epithelium ((**C**), arrow). H&E stain, the bar size is indicated under pictures ((**A**,**B**) = 100 µm, (**C**) = 20 µm).

**Figure 13:**
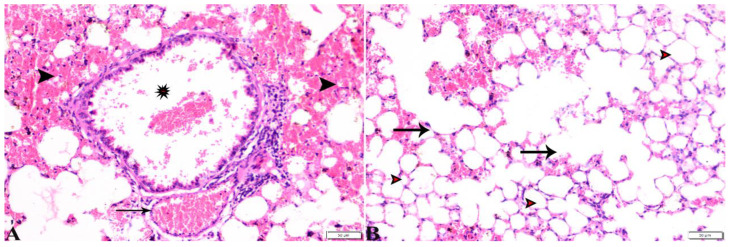
Photomicrograph of lung tissue sections from *Toxoplasma gondii*-infected group (G3) treated with Propolis showing: (**A**) Congested blood vessel (arrow), dilated bronchiole with degenerated epithelium (star), peribranchial and interstitial hemorrhage (arrowheads). (**B**) Collapsed alveoli (arrowheads), some alveoli showing compensatory emphysema (arrows). H&E stain, the bar size is indicated under pictures ((**A**,**B**) = 50 µm).

**Figure 14:**
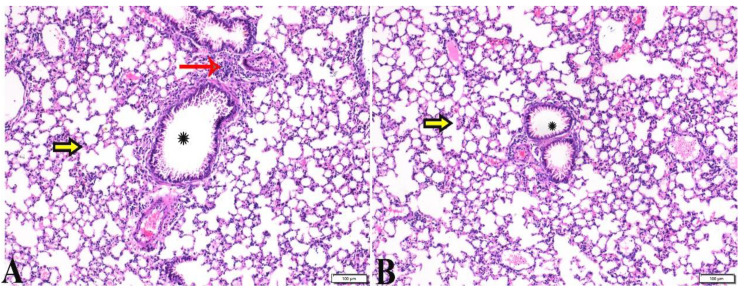
Photomicrograph of lung tissue sections from *Toxoplasma gondii*-infected group treated with wheat germ oil (G4) showing normal bronchiolar structure ((**A**,**B**), stars), normal alveolar tissue ((**A**,**B**), yellow arrows), and normal peribranchial lymphoid cellular structure ((**A**), red arrow). H&E stain, the bar size is indicated under pictures.

**Figure 15:**
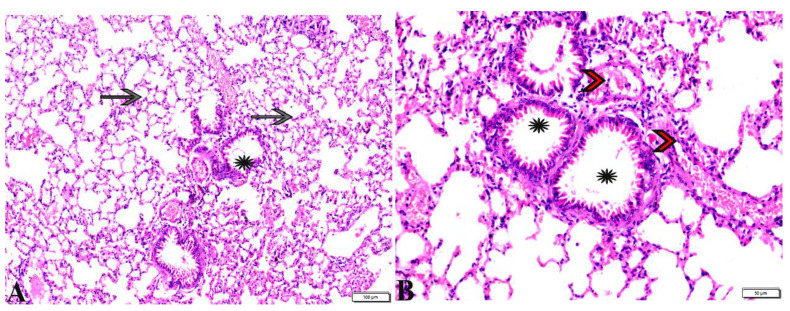
Photomicrograph of lung tissue sections from combination (Propolis and wheat germ)-treated group (G5) showing normal bronchiolar structure ((**A**,**B**), stars), normal alveolar tissue ((**A**), arrows), and mild congestion ((**B**), red arrow heads). H&E stain, the bar size is indicated under pictures.

**Figure 16:**
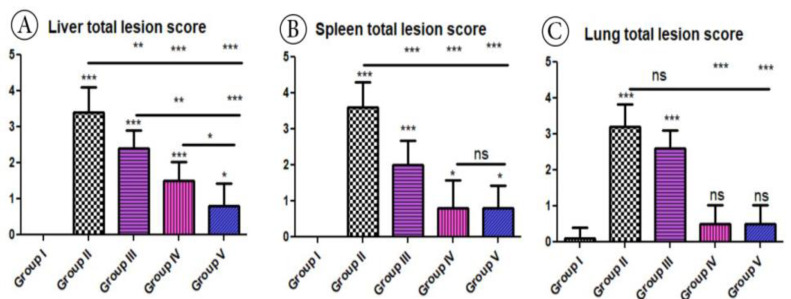
Histomorphometry graph showing quantitative and semiquantitative measurements of total lesion scores recorded in tissue sections among the experimental groups: (**A**) Liver total lesion score, (**B**) Spleen total lesion score and, (**C**) Lung total lesion score. Data are expressed as means ± standard deviations. Significant differences vs. the control group are marked by different asterisks through one-way ANOVA with Tukey’s post hoc test: * *p* ≤ 0.05, ** *p* ≤ 0.01, *** *p* ≤ 0.001, ns Non significant.

The histopathological images of the liver, spleen, and lungs, tissue specimens are shown in Figure 2, Figure 3, Figure 4, Figure 5, Figure 6, Figure 7, Figure 8, Figure 9, Figure 10, Figure 11, Figure 12, Figure 13, Figure 14, Figure 15 and Figure 16. As shown in Figure 2, the liver tissue obtained from the normal healthy mice (negative control, G1) expressed a normal hepatic architecture, comprising a normal central vein (C.V), sinusoids (S), and hepatocytes (H) besides normal portal triad structures (hepatic artery, portal vein (P.V), and a normal bile duct. The sections obtained from infected mice (positive control group, G2) showed several histopathological changes, including severe dilatation in C.V, and the presence of bradyzoites between the hepatic tissue and inside the paracentral tissue surrounded by mononuclear cellular infiltration. In addition, the presence of focal areas of hepatocellular necrosis, the proliferative reaction of Kupffer cells, and marked periportal edema around P.V were also noticed (Figure 3). 

Table 3 treated with propolis showed a normal (C.V) and marked improvement in hepatocellular histological structure, but the tachyzoites were present between hepatic tissues, and tissue cysts were present (Figure 4). The histopathological structure of the liver tissue in G4, which was treated with WGO, expressed a normal central vein (C.V) and the presence of a mild periportal inflammatory cellular reaction; however, there was a proliferative reaction of Kupffer cell reaction, a dilated P.V, collection of some bradyzoites in hepatocytes and a focal area of coagulative hepatocellular necrosis appeared (Figure 5). In G5, which was treated with the propolis and WGO in combination, there was an improvement in the liver tissue section (Figure 6), which was expressed by the presence of a normal C.V, and normal P.V. However, there was mild vacuolar degeneration in the hepatocyte structure, mild focal mononuclear cellular aggregation between hepatic tissues, and mild periportal mononuclear cellular infiltration.

Regarding the histopathological changes in the spleen tissue, Figure 7 expresses the normal histological structure of the spleen tissue obtained from the non-infected mice (G1), which exhibited a normal splenic artery, normal-sized white pulp with distinct cellular density in the germinal center and a normal red pulp. On the other hand, the sections from spleen tissue of the positive control group (G2) revealed severe depletion in the lymphocyte population in the splenic white and red pulp. The existence of marked numbers of megakaryocytes between multiple bradyzoites was noted, in addition to the presence of a single *T. gondii* tachyzoite and multiple bradyzoites, as well as the diffuse mononuclear leucocytic cellular infiltration (Figure 8). Splenic tissue sections obtained from animals treated with propolis (G3) expressed a near normal-sized splenic white pulp, haemorrhage in the red pulp, and the presence of some megakaryocytes surrounded by multiple bradyzoites (Figure 9).

Interestingly, the normal histological picture of the splenic tissue sections, obtained from animals treated with WGO (G4), expressed a normal-sized splenic white pulp and normal red pulp (Figure 10). Furthermore, a noticeable improvement was observed in splenic tissue sections obtained from G5, which was treated with both propolis and WGO, as shown in Figure 10. Moreover, a normal-sized splenic white pulp and normal red pulp were observed, and there were some megakaryocytes. The microscopic examination of the lung tissue obtained from the negative control group (G1) revealed a normal bronchiolar structure and normal alveolar tissue (Figure 11). In contrast, the lung tissue obtained from the positive control group (G2) showed congested blood vessels, atelectasis in alveoli with mild interstitial haemorrhage, and hyperplasia in the bronchial epithelium (Figure 12). The lung tissue sections obtained from infected animals treated with propolis (G3) exhibited congested blood vessels, dilated bronchioles with a degenerated epithelium, peribranchial and interstitial haemorrhage, collapsed alveoli, and some alveoli showing compensatory emphysema (Figure 13) Whereas a normal histological image was observed in the lung tissue obtained from G4 treated with WGO (Figure 14). A normal bronchiolar structure, alveolar tissue, and peribranchial lymphoid cellular structure have appeared. In addition, as shown in Figure 15, lung tissue obtained from the group treated with propolis and WGO (G5) showed normal bronchiolar structure, alveolar tissue, and peribranchial lymphoid cellular structure. Certainly, G5 revealed a normal histological image in the lung tissue specimens (Figure 15).

Based on histopathological scoring, the severity of parasitic tissue cyst number in liver tissue, dilatation in C.V, hepatocellular necrosis, and the inflammatory cellular reaction showed significant (*p* ≤ 0.05) improvement in all treated groups compared to the positive control group (Figure 16). In the same context, the quantitative scoring of the parasitic tissue cyst number, number of megakaryocytes, mononuclear leucocytic cellular infiltration, and the size of white and red pulp of the spleen displayed significant (*p* ≤ 0.05) improvements among all the experimental groups compared to the positive control group. In addition, based on histopathology scoring (Figure 16) of the lungs, the severity of parasitic tissue cyst number, interstitial haemorrhage, blood vessel congestion, and bronchiolar epithelium structure was significantly improved (*p* ≤ 0.05) in all treated experimental groups (G3, G4, and G5) compared to the positive control group (G2).

## 4. Discussion

As mentioned above, most drugs that act against toxoplasmosis, such as pyrimethamine–sulfadiazine, have many side effects. Several recent studies have combined chemical drugs with natural compounds, such as propolis, in order to enhance their synergistic effects [65]. In a previous research, we reported the potential benefits of WGO and propolis combination in the treatment of chronic toxoplasmosis in mice [17,18]. However, no previous work was conducted on their use against acute toxoplasmosis. 

It is noteworthy that propolis has many plant secondary metabolite contents (phenolic compounds) that work against many extracellular and intracellular protozoan parasites. A previous study reported its anti-trypanosomal effect by stimulating cell lysis and reducing the number of pathogen lipids [66]. Furthermore, these phenolics promoted cytoplasmic condensation and DNA aggregation in *Leishmania Donovani* [67]. Another study [34] reported that propolis achieved up to 70% reduction in parasitemia in mice infected with *Plasmodium chabaudi*. An additional study showed the antiprotozoal activity of propolis against *Leishmania* and *Trichomonas vaginalis* [32]. In the present study, the efficacy of the combined use of propolis and WGO could be roughly estimated through assessing the parasite load parasitologically, molecularly, and histopathologically. According to our findings, propolis can significantly reduce the parasite load in the examined organs, revealing that the stained tissue smears (liver) obtained from the animals treated with propolis exhibited a significant reduction in the parasite load compared to the positive control group. This result agrees with a previous work [51] that reported that propolis extract alone can reduce the average brain parasite load during chronic toxoplasmosis by 16.71%. Furthermore, in the same study [51], the propolis extract achieved a higher reduction percentage (31.74%) when combined with spiramycin, potentiating the therapeutic activity of spiramycin. One more study proved the antiprotozoal activity of propolis since it inhibits tachyzoite multiplication, and its antiparasitic effect could be related to the presence of caffeic acids, terpenoids, and flavonoids [33]. 

Additionally, the present results agree with a previous study [28] that reported the synergetic effect of propolis in combination with spiramycin against acute toxoplasmosis with a reduction percentage of >96% in parasitic load in the liver, spleen, and brain through enhancing the ability of spiramycin to penetrate the blood–brain barrier. In the present work, the results of RT-PCR confirmed the parasitological results since there was a significant reduction in the parasite load in the animals treated with propolis (G3) compared to the positive control group (G2), which had the highest parasite load. The present results are in harmony with previous works that revealed the antiparasitic activity of propolis and WGO in treating chronic toxoplasmosis in mice [17,18]. 

Moreover, the histopathological examination and scoring of the liver, spleen, and lungs confirmed the previously achieved results. In this context, there was marked cellular damage in the liver, spleen, and lung tissues of the positive control group (infected–untreated). This result could be attributed to the potency of *T. gondii* tachyzoites for invading and rupturing any nucleated cell; in addition, they strongly stimulate cytokine release, which has a lytic effect on the infected cells [68,69]. The histopathological changes in the liver tissue specimens obtained from the positive control group revealed dilations in the central vein and hepatocellular necrosis, in addition to the presence of bradyzoites between the hepatic tissue and inside the paracentral tissue surrounded by mononuclear cellular infiltration, which is in agreement with some previous works against chronic toxoplasmosis [17,18]. Furthermore, the present study reported the proliferative reaction of Kupffer cells and marked periportal edema around the portal vein. Similar histopathological changes in the liver tissue of mice intraperitoneally infected with *T. gondii* tachyzoites were reported in another study that recorded severe inflammatory infiltration in the portal track, marked dilation in the central and portal veins, and the presence of parasites inside and outside the hepatic cells [28]. Our histopathological findings are also consistent with previous studies [17,18,69]. The histopathological changes in the spleens of untreated animals showed congested white and red pulp and the presence of mononuclear cell infiltration and megakaryocytes, which agrees with some previous works [17,18]. This damage might result from the stimulation of tachyzoites by inflammatory cells, which release a high level of IL12 [70].

The present work concluded obvious improvement of the histopathological changes in the liver obtained from animals treated with propolis expressed in the absence of mononuclear cell infiltration, hepatocellular necrosis, and peritoneal edema, and this result is consistent with previous works [17,18]. With respect to the spleen, a clear improvement was observed in the spleen tissue of the animals treated with propolis. In the present work, the white and red pulp had a normal size, and there was a reduction in megakaryocyte number, but hemorrhage was still present. These findings are in line with a previous study [28], which reported an obvious improvement in the liver and spleen of animals treated with a combination of propolis and spiramycin. The reduction in the inflammatory process in affected organs may be attributed to the anti-inflammatory activity of propolis, which decreased the production of monokines and interferon, in addition to its ability to inhibit lymphocyte proliferation [71]. A minor improvement in lung tissue obtained from animals treated with propolis (G3) was also observed in the present study. However, according to the histopathological scoring, there was a significant (*p* ≤ 0.05) improvement in the G3 group treated with propolis compared to the positive control group in all organs. It should be stressed that WGO is a vegetable oil with anti-inflammatory and antioxidant properties [37,38]. A previous study [72] reported that fermented wheat germ extract stimulates cell apoptosis and inhibits the proliferation of cancer cells. Regarding its antiparasitic effects, wheat germ extract reported antiparasitic activity against *Giardia lamblia* in combination with probiotics [73]. Another study [74] reported an anti-inhibitory effect on the growth of *G. lamblia* trophozoites. Furthermore, a study demonstrated that wheat germ agglutinin extract has an obvious inhibitory effect on the motility and growth of the *Trichomonas vaginalis* trophozoite [42]. 

Furthermore, WGO has antiparasitic activity against cryptosporidiosis [41]. In the present study, the effect of WGO was first investigated against *T. gondii*, and it reduced the parasite load (*p* < 0.05). The parasitological assessment of the parasite load in the examined organs revealed that the stained tissue smears obtained from the animals treated with WGO (G3) exhibited a significant reduction in the parasite load compared to the parasite load in the positive control group, which has been reported elsewhere [17,18]. In the same line, the results of RT-PCR confirmed the parasitological results, as there was a significant reduction in the parasite load in the animals treated with WGO (G3) compared to the positive control group (G2), which had the highest parasite load. The histopathological examination of liver tissue specimens revealed little improvement in the pathological picture of the liver tissue obtained from animals treated with WGO, as there was still an inflammatory cellular reaction, dilated portal vein and focal areas of coagulative hepatocellular necrosis. In addition, an improvement occurred in the spleen tissue of the treated animals with WGO (G4), whereas the white and red pulp had a normal size with a reduction in megakaryocyte number. Furthermore, a marked improvement in the histological picture of lung tissue was observed in the same group (G4). Our findings demonstrated that WGO treatment improved the histopathological picture of the affected organs. These findings agree with a previous study [41], which reported an obvious improvement in the histopathological picture of ileocecal sections obtained from animals treated with WGO against cryptosporidiosis infection. In the present study, those animals treated with the combination of propolis and WGO (G5) achieved the best parasitological and molecular results and had the lowest parasitic load when compared to other treatment groups. In addition, both treatments improved the pathological picture of the infected organs and restored the histopathological alterations in the liver, spleen and lungs. Similar findings were reported due to the activity of propolis and WGO against chronic toxoplasmosis in mice [17,18]. 

## 5. Conclusions

In conclusion, the present work revealed the potential synergistic effects of propolis and WGO of herbal substance used in this study, which could be considered an effective treatment of acute toxoplasmosis infection. These herbal substances reduced the parasite load significantly in the liver, spleen and lungs. In addition, they markedly improved the histopathological picture of these organs. Further future research is warranted to explore more about the mechanistic pathways underlying these effects of propolis and WGO against acute toxoplasmosis.

## Figures and Tables

**Table 1:** The experimental protocol and groups.

Group Order	Treatment Strategy
Group 1 (G1)	Negative control group (non-infected and non-treated)
Group 2 (G2)	Positive control group (infected non-treated group)
Group 3 (G3)	Infected mice treated with 0.1 mL propolis extract/day [17,18,51]
Group 4 (G4)	Infected mice treated with WGO at a dose of 0.2 mg/1.5 mL/kg bw/day [17,18,52].
Group 5 (G5)	Infected mice treated with a combination of propolis and WGO at the same mentioned doses.

**Table 2:** Oligonucleotides used for real-time polymerase chain reaction (RT-PCR).

P29 Q-f	CAGCATGGATAAGGCATCTG
P29 Q-r	GTTGCTCCTCTGTTAGTTCC

**Table 3:** The CT values and absolute quantity of both standard and treated samples.

Experiment	Group	Group Type	Ct (dRn)	Quantity (Copies)
NTC	G1	NTC (non-infected- non treated)	No Ct	No Ct
Positive Control (G2)	G2-a	Standard infected non treated	21.69	6.40 × 10^−0^
	G2-b	Standard infected non treated	21.63	6.50 × 10^−0^
	G2-c	Standard infected non treated	25.06	6.50 × 10^−0^
	G2-d	Standard infected non treated	22.1	6.40 × 10^−0^
	G2-e	Standard infected non treated	21.53	6.40 × 10^−0^
Treated Groups	G3-a	Infected Treated with Propolis	24.7	5.61 × 10^−1^
	G3-b	Infected Treated with Propolis	26.54	5.23 × 10^−1^
	G3-c	Infected Treated with Propolis	26.02	6.21 × 10^−1^
	G3-d	Infected Treated with Propolis	25.21	5.62 × 10^−1^
	G3-e	Infected Treated with Propolis	23.98	5.71 × 10^−1^
	G4-a	Infected Treated with Wheat germ oil	31.89	3.71 × 10^−2^
	G4-b	Infected Treated with Wheat germ oil	29.39	4.43 × 10^−1^
	G4-c	Infected Treated with Wheat germ oil	30.4	5.13 × 10^−1^
	G4-d	Infected Treated with Wheat germ oil	29.01	6.42 × 10^−2^
	G4-e	Infected Treated with Wheat germ oil	29.68	4.36 × 10^−2^
	G5-a	Infected Treated with combination of Wheat germ oil and Propolis.	34.74	3.05 × 10^−3^
	G5-b	Infected Treated with combination of Wheat germ oil and Propolis.	32.7	3.62 × 10^−1^
	G5-c	Infected Treated with combination of Wheat germ oil and Propolis.	36.85	3.42 × 10^−1^
	G5-d	Infected Treated with combination of Wheat germ oil and Propolis.	37.03	2.51 × 10^−1^
	G5-e	Infected Treated with combination of Wheat germ oil and Propolis.	37.42	2.33 × 10^−2^

‘’a–e’’, refers to the serial number of the sample withing the group.

**Table 4:** The variable change in the *T. gondii* load in liver after treatment with the herbal substances compared to the control drug-treated, infected, and non-treated animals.

Sample ID	Quantity
C-(G2;Infected untreated) sample	0.00 × 10 + 00
S1 (G3; Treated with Propolis)	5.84 × 10^−1^
S2 (G4; Treated with Wheat germ oil)	4.78 × 10^−2^
S3 (G5:Treated with combination of Wheat germ oil and Propolis)	2.97 × 10^−2^

## Data Availability

Not applicable.
